# Esophageal Hematoma After Severe Vomiting

**DOI:** 10.7759/cureus.74392

**Published:** 2024-11-25

**Authors:** Jiaming Lei, Ling Wu

**Affiliations:** 1 Department of Gastroenterology, People’s Hospital of Leshan, Leshan, CHN; 2 Department of Cardiology, The Affiliated Hospital of Southwest Medical University, Luzhou, CHN

**Keywords:** chest pain, esophageal hematoma, esophageal injury, hematemesis, vomit

## Abstract

A male patient developed hematemesis and chest pain after severe vomiting. Gastroscopy showed a linear hematoma from the esophageal entrance to the cardia. Enhanced CT of the esophagus revealed a high-density shadow in the middle of the esophagus. Severe vomiting can lead to esophageal injury and esophageal hematoma. As this condition mimics critical symptoms of myocardial infarction and aortic dissection, clinicians must remain vigilant. This case emphasizes the importance of recognizing acute submucosal bleeding in the digestive tract, aiding in clinical diagnosis and treatment.

## Introduction

Esophageal hematoma is an uncommon but significant esophageal injury characterized by submucosal bleeding and hematoma formation, often secondary to trauma or increased intraesophageal pressure [1,2]. It can be caused by various factors, including coagulopathy, foreign body ingestion, severe vomiting, or endoscopic interventions [3]. While rare, esophageal hematomas can cause severe symptoms such as chest pain, hematemesis, and dysphagia, which are easily mistaken for myocardial infarction or aortic dissection, leading to potential misdiagnosis [4]. Diagnosing esophageal hematoma requires a detailed review of clinical history, endoscopic examination, and imaging studies [5].

Unlike mucosal tears, which occur when the esophageal lesion breaches the mucosal layer, or esophageal ruptures, where the lesion breaches the muscular layer, most esophageal hematomas remain confined within the submucosa, without obvious breakthroughs in either direction. This characteristic is key to the often extensive range of hematoma observed in clinical cases and is closely linked to the pathophysiological mechanism of the condition.

This report presents a case of esophageal hematoma caused by severe vomiting, highlighting its clinical features, diagnostic process, and treatment. The findings underscore the necessity of differentiating this condition from other life-threatening esophageal and cardiovascular disorders.

## Case presentation

**Figure 1:**
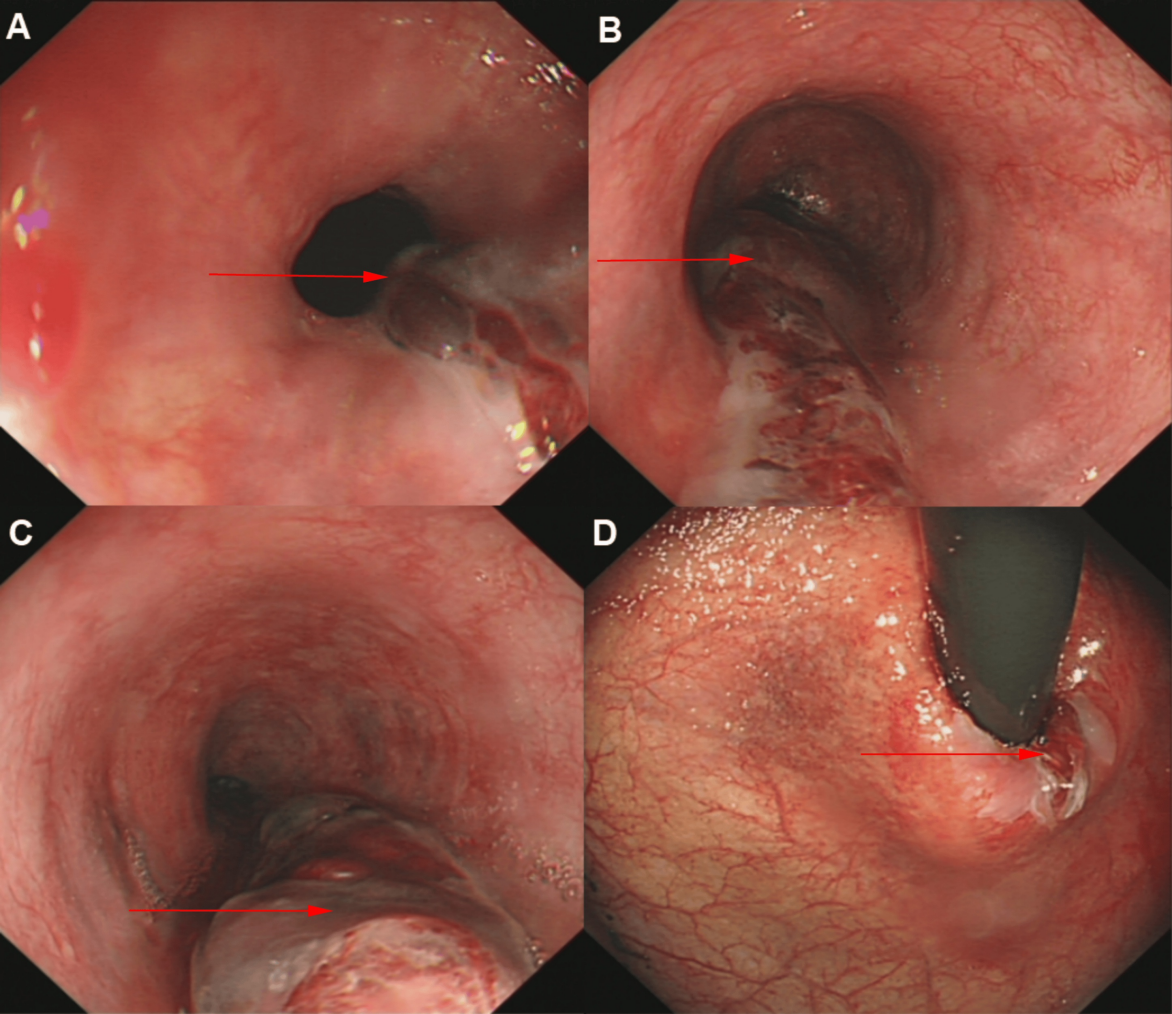
Esophageal hematoma in whole esophagus (red arrow); (A) esophageal entrance, (B) middle esophagus, (C) lower esophagus, and (D) cardia.

A 71-year-old healthy male experienced nausea after eating fish, followed by a single episode of severe vomiting with a large amount of food material. He subsequently developed intense chest pain and vomited fresh blood four times, totaling approximately 100 mL. On examination, the patient appeared mildly distressed with stable vital signs. His abdomen was soft and non-tender, with no guarding or rebound tenderness. Cardiovascular and respiratory examinations were unremarkable. The patient denied foreign body sensation, referred pain, black stools, dyspnea, chest tightness, palpitations, or fever. He had a five-year history of well-controlled diabetes and no liver disease or cirrhosis. There was no history of gastrointestinal bleeding. He sought emergency care. Laboratory results showed a hemoglobin level of 112 g/L, with no significant abnormalities in cardiac troponins, myocardial enzymes, coagulation tests, or electrocardiogram. Chest CT revealed esophageal wall thickening without evidence of a foreign body. Gastroscopy identified a 1.0 cm linear hematoma extending from the esophageal entrance to the cardia, dark red in color, located on the left esophageal wall. It was associated with surface erosion and slight blood oozing, with high tension in the superficial tissue layer (Figure 1A-1D). Gastric mucosal atrophy was also noted.

**Figure 2:**
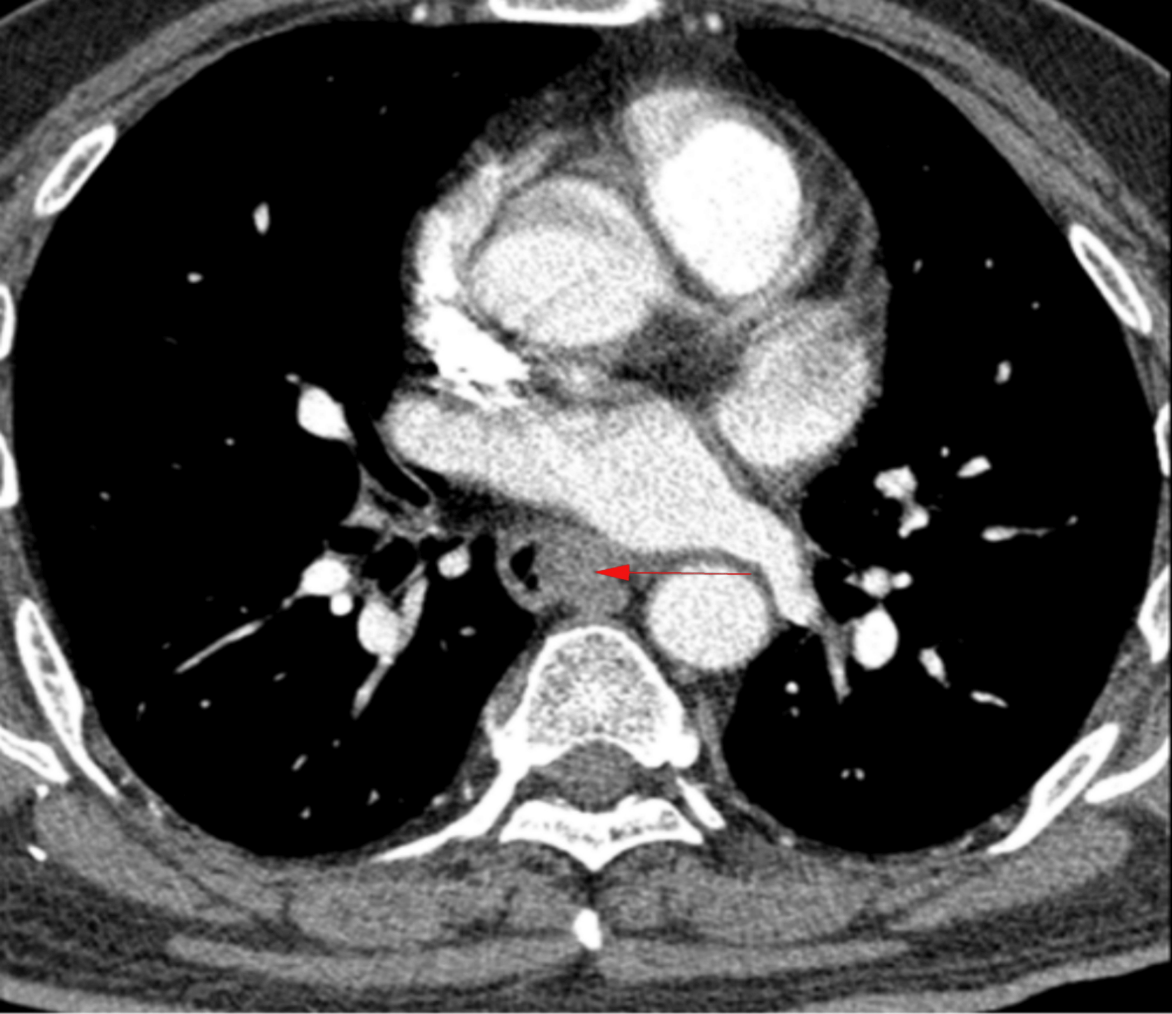
A strip of slightly high-density shadow on the left side of the mid-esophageal lumen in enhanced thoracic esophageal CT (red arrow).

A contrast-enhanced thoracic esophageal CT revealed a slightly higher density strip-like shadow on the left side of the esophageal lumen at the T6-T7 vertebra level, causing eccentric narrowing. The thickest part measured approximately 1.5 cm, with a CT value of 43 HU, and no significant enhancement after contrast (red arrow, Figure 2). No free gas was detected in the mediastinum.

**Figure 3:**
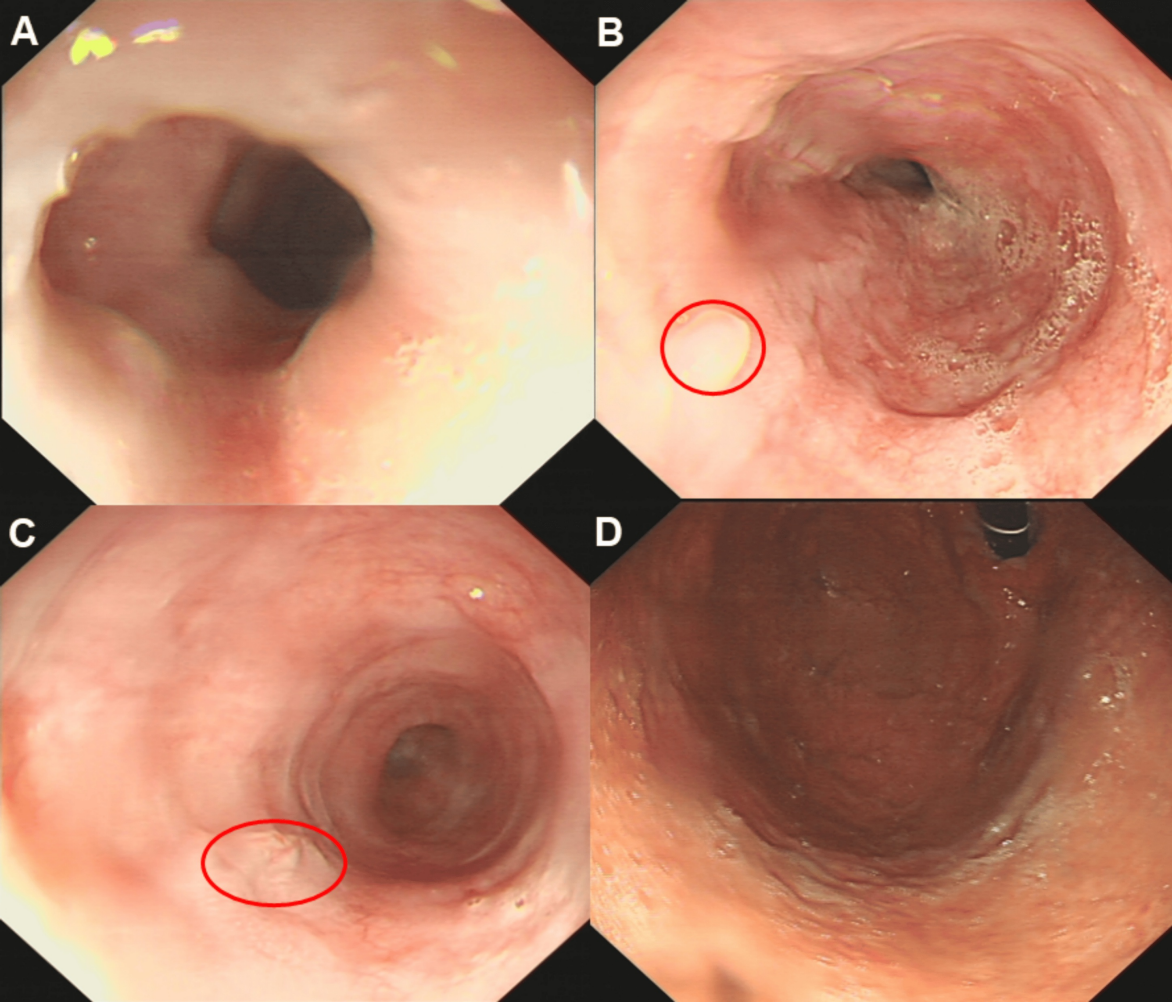
A follow-up gastroscopy one month later showed complete recovery, with no residual hematoma and healed mucosa, leaving a white scar (oval circle); (A) esophageal entrance, (B) middle esophagus, (C) lower esophagus, and (D) fundus of stomach.

Based on the patient's history, symptoms, clinical examination, laboratory results, imaging, and endoscopic findings, the diagnosis of an esophageal wall hematoma was confirmed. The patient was managed conservatively with fasting, nutritional support, and symptomatic pain relief, and was discharged after improvement. A follow-up gastroscopy one month later showed complete recovery, with no residual hematoma and healed mucosa, leaving a white scar (oval circle, Figure 3).

## Discussion

Esophageal hematoma is a rare manifestation of esophageal injury, typically associated with acute chest pain, hematemesis, and vomiting. These symptoms overlap with life-threatening conditions such as myocardial infarction, aortic dissection, and pulmonary embolism, necessitating careful differential diagnosis [1,6]. In this case, gastroscopy and imaging ruled out foreign body injury and full-thickness esophageal rupture (Boerhaave syndrome), confirming the diagnosis of esophageal hematoma.

In terms of pathophysiology, severe vomiting induces vigorous esophageal contractions and peristalsis, leading to submucosal tearing and bleeding. The submucosal layer, composed of loose connective tissue, continues to bleed, but the blood does not penetrate the esophageal lumen or extraluminal spaces. This increases the volume and pressure within the submucosa, causing greater tensile force. The heightened tension further disrupts the submucosal layer, separating it from adjacent mucosal and muscular layers. This "zipper-like" chain reaction results in new submucosal vascular ruptures and bleeding, ultimately forming an extensive longitudinal hematoma along the esophagus, as observed in this case.

Unlike mucosal tears or full-thickness esophageal ruptures, most esophageal hematomas do not breach the mucosal or muscular layers. This containment within the submucosal space accounts for the extensive hematoma observed in many cases and is directly linked to the pathophysiological mechanism of progressive bleeding and tissue separation.

There is limited data regarding the downward growth of esophageal hematomas, but previous cases suggest that the hematoma may extend longitudinally due to the mechanical forces exerted during vomiting and esophageal peristalsis [5]. Unlike mucosal or muscular ruptures, the lack of significant breakthroughs in esophageal hematomas highlights their distinct pathology and potential for extensive spread along the submucosal layer.

The causes of esophageal hematoma include coagulopathy, trauma, foreign body ingestion, endoscopic procedures, and forceful vomiting [6-8]. Severe vomiting, as experienced by this patient, can result in different depths of mucosal injury, ranging from Mallory-Weiss tears to submucosal hematomas. Unlike Mallory-Weiss syndrome, which involves superficial mucosal tears, esophageal hematomas are confined to the submucosal layer and do not involve transmural rupture.

Management is typically conservative, involving fasting, nutritional support, and symptomatic treatment [6,8]. Surgical intervention is rarely required and reserved for complications or significant obstruction. This case, along with previous reports, highlights the efficacy of conservative treatment, demonstrated by the patient’s complete recovery and resolution of symptoms.

## Conclusions

Esophageal hematoma is a rare but clinically significant condition that must be considered in patients presenting with acute chest pain, hematemesis, and vomiting. Various causes, such as severe vomiting, coagulopathy, or trauma, should be considered in differential diagnosis. Accurate diagnosis requires integrating clinical history, imaging, and endoscopic findings to differentiate it from other critical conditions. Conservative management, including fasting, nutritional support, and symptomatic treatment, is effective in most cases, as demonstrated by the patient’s successful recovery. Further studies are necessary to better understand its pathophysiology and treatment strategies.
